# The serological immunogenicity of the third and fourth doses of COVID-19 vaccine in patients with inflammatory rheumatic diseases on different biologic or targeted DMARDs: a Swedish nationwide study (COVID-19-REUMA)

**DOI:** 10.1128/spectrum.02981-23

**Published:** 2024-03-05

**Authors:** Martina Frodlund, Per Nived, Katerina Chatzidionysiou, Anna Södergren, Eva Klingberg, Monica Hansson, Sophie Ohlsson, Elisa Pin, Anders Bengtsson, Lars Klareskog, Meliha Kapetanovic

**Affiliations:** 1 Department of Biomedical and Clinical Sciences, Division of Inflammation and Infection/Rheumatology, Linköping University, Linköping, Sweden; 2 Department of Clinical Sciences, Section for Rheumatology, Lund University, Lund and Skåne University Hospital, Lund, Lund, , Sweden; 3 Department of Medicine, Rheumatology Unit, Karolinska University Hospital and Karolinska Institutet, Stockholm, Solna, Sweden; 4 Department of Public Health and Clinical Medicine/Rheumatology, Umeå University, Umeå, Sweden; 5 Department of Rheumatology and Inflammation Research, Sahlgrenska Academy, University of Gothenburg, Gothenburg, Sweden; 6 Department of Clinical Sciences, Section for Nephrology, Lund University, Lund and Skåne University Hospital, Lund, Lund, , Sweden; 7 Department of Protein Science, SciLifeLab, KTH Royal Institute of Technology, Stockholm, Sweden; National Chung Hsing University, Taichung, Taiwan

**Keywords:** COVID-19 vaccine, serological response, biological/targeted DMARDs, response rate, omicron subvariants, immunogenicity, long-term immunogenicity

## Abstract

Studies investigating the immunogenicity of additional COVID-19 vaccine doses in immunosuppressed patients with inflammatory rheumatic diseases (IRD) are still limited. The objective was to explore the antibody response including response to omicron virus subvariants (sBA.1 and sBS.2) after third and fourth COVID-19 vaccine doses in Swedish IRD patients treated with immunomodulating drugs compared to controls. Antibody levels to spike wild-type antigens (full-length protein and S1) and the omicron variants sBA.1 and sBA.2 (full-length proteins) were measured. A positive response was defined as having antibody levels over cut-off or ≥fourfold increase in post-vaccination levels for both antigens. Patients with arthritis, vasculitis, and other autoimmune diseases (*n* = 414), and controls (*n* = 61) receiving biologic/targeted synthetic disease-modifying anti-rheumatic drugs (DMARDs) with or without conventional synthetic DMARDs participated. Of these, blood samples were available for 370 patients and 52 controls after three doses, and 65 patients and 15 controls after four doses. Treatment groups after three vaccine doses were rituximab (*n* = 133), abatacept (*n* = 22), IL6r inhibitors (*n* = 71), JAnus Kinase inhibitors (JAK-inhibitors) (*n* = 56), tumor necrosis factor inhibitor (TNF-inhibitors) (*n* = 61), IL12/23/17 inhibitors (*n* = 27), and controls (*n* = 52). The percentage of responders after three and four vaccine doses was lower in rituximab-treated patients (59% and 57%) compared to controls (100%) (*P* < 0.001). After three doses, the percentage of responders in all other groups was 100%, including response to omicron sBA.1 and sBA.2. In rituximab-treated patients, higher baseline immunoglobulin G (IgG) and longer time-period between rituximab and vaccination predicted better response. In this Swedish nationwide study including IRD patients three and four COVID-19 vaccine doses were immunogenic in patients treated with IL6r inhibitors, TNF-inhibitors, JAK-inhibitors, and IL12/23/17-inhibitors but not in rituximab. As >50% of rituximab patients responded to vaccines including omicron subvariants, these patients should be prioritized for additional vaccine doses.

**IMPORTANCE:**

Results from this study provide further evidence that additional doses of COVID-19 vaccines are immunogenic and result in satisfactory antibody response in a majority of patients with inflammatory rheumatic diseases (IRD) receiving potent immunomodulating treatments such as biological or targeted disease-modifying anti-rheumatic drugs (DMARDs) given as monotherapy or combined with traditional DMARDs. We observed that rituximab treatment, both as monotherapy and combined with csDMARDs, impaired antibody response, and only roughly 50% of patients developed a satisfactory antibody response including response to omicron subvariants after the third vaccine. In addition, higher IgG levels at the last rituximab course before the third vaccine dose and a longer time after the last rituximab treatment increased the chance of a satisfactory antibody response. These results indicate that rituximab-treated patients should be prioritized for additional vaccine doses.

**CLINICAL TRIALS:**

EudraCT (European Union Drug Regulating Authorities Clinical Trials Database) with number 2021-000880-63.

## INTRODUCTION

Infectious diseases constitute an important cause of morbidity and mortality in patients with inflammatory rheumatic diseases (IRDs) (1, 2). Infectious complications in these patients are most likely the result of a combination of immunopathology of the IRD itself, associated comorbidities, and immunosuppressive anti-rheumatic treatment (3).

The severe acute respiratory syndrome coronavirus-2 (SARS-CoV-2) pandemic has to date resulted in over 764 million confirmed cases of COVID-19 disease, and according to the WHO an estimated 22 million in excess mortality from January 2020 through April 2023 (4).

Several studies have shown increased risks of hospitalization, following COVID-19 infection in patients with IRD (5–8). A nationwide Danish cohort study showed 72% and 82%, respectively, higher risks of hospitalization for people with rheumatoid arthritis (RA) and vasculitides, compared to the general population (5). Although a subject of debate, studies from South Korea, Sweden, and the UK, showed a higher risk of death after COVID-19 in people with IRD compared to the general population (6, 7, 9, 10). These results were supported by a meta-analysis which identified an increased mortality rate (OR 1.74) for patients with COVID-19 infection and rheumatic and musculoskeletal disease (RMD) compared to patients without RMD (11). Age, comorbidities, glucocorticoid use, B cell depleting therapy with anti-CD20 (rituximab), and disease activity have been associated with COVID-19-related death in patients with IRD (12).

Vaccination is the most important preventive measure against severe COVID-19 disease. Three COVID-19 vaccines were introduced in Sweden in early 2021, i.e., two mRNA vaccine platforms (BNT162b2 and mRNA-1273), and one adenovirus vector vaccine (ChAdOx1 nCoV-19) (13–15). The immunogenicity, efficacy, and safety of these vaccines have been demonstrated (13–15). All adults were recommended a primary series of two vaccine doses, and in August 2021 the Public Health Agency of Sweden published updated recommendations on the use of an extra dose at least 8 weeks after the second dose to immunocompromised persons (16). Waning immunity within months after COVID-19 vaccination in healthy adults and the emergence of the omicron variant led to the recommendation of a third vaccine dose to all adults in Sweden, in December 2021, supported also by the European Alliance of Associations for Rheumatology (17). A recent study has shown accelerated waning in patients with immune-mediated inflammatory diseases (IMIDs) three months after COVID-19 vaccination compared to healthy controls (18). New recommendations from the Public Health Agency of Sweden in March 2023 advocated two COVID-19 vaccine doses a year for immunocompromised individuals and elderly (≥80 years) and once per year for people 65–79 years or individuals with risk factors for serious COVID-19 infection (15).

Our group has previously reported that the humoral immune response following two vaccine doses against COVID-19 is severely impaired with rituximab treatment, reduced with abatacept, and to some extent also reduced with interleukin-6 receptor inhibitors (IL6ri) in patients with IRD compared to controls (19). Only a few patients developed satisfactory antibody response when vaccinated less than 6 months after the last dose of rituximab, which is in line with other studies of patients with IRD reporting below 20% response rate for patients treated with rituximab within 6 months prior to vaccination (20, 21).

In a Norwegian study of patients with IMIDs, participants with poor antibody response after two doses of the COVID-19 vaccine (10%), received a third dose resulting in an 84% response rate (22). Poor responders were mainly patients treated with tumor necrosis factor inhibitor (TNFi) in combination with methotrexate or azathioprine therapy, JAnus Kinase inhibitors (JAKi) or abatacept, but the study did not include patients with rituximab. Another study from this group included RA patients treated with rituximab, and although a third vaccine dose only induced a serological response in 16% of patients, CD4^+^ and CD8^+^ T cell responses were seen in all assessed patients (23). Benucci et al. studied the effect of a third dose in 200 RA patients vaccinated with the BNT162b2 mRNA vaccine and found a negative influence on seroconversion particularly for patients treated with abatacept or rituximab (24).

The aims of this study were to investigate (i) if a third and fourth dose SARS-CoV-2 vaccine improves serological responses, compared to two doses, in IRD patients with biologic or targeted synthetic Disease Modifying Anti-Rheumatic Drug (b/tsDMARD) treatment given as monotherapy or in combination with conventional synthetic DMARDs (csDMARDs) compared to healthy controls; (ii) the serological response to omicron subvariants sBA.1 and sBA.2 measured after the third vaccine dose in these patients.

## MATERIALS AND METHODS

The present study was conducted at five University Rheumatology departments in five different regions across Sweden: Region Västerbotten, Region Stockholm, Region Östergötland, Västra Götalandsregionen, and Region Skåne.

### Patient population

Patients with IRD with regular follow-up at the rheumatology departments and receiving treatment with b/tsDMARDs were offered to participate. The disease and treatment characteristics of the total patient populations are described previously (19) and corresponding data at the third vaccine dose are provided in Table 1. The b/tsDMARDs therapies evaluated were (rituximab), JAKi (tofacitinib, baricitinib, and upadacitinib), TNFi (infliximab, adalimumab, etanercept including the biosimilars of these, certolizumab pegol, and golimumab), IL6ri (tocilizumab, sarilumab), T cells co-stimulation inhibitor (abatacept), IL12/23i (ustekinumab), and IL17i therapy (sekukinumab, ixekizumab). These treatments were administrated as monotherapy or in combination with other csDMARDs. Patients with the following rheumatic diseases participated: rheumatoid arthritis (RA), spondylarthritis including psoriatic arthritis, juvenile idiopathic arthritis (JIA) and other arthritides, patients with systemic vasculitis including antineutrophilic cytoplasmic antibody associated systemic vasculitis and giant cells arteritis as well as patients with other inflammatory diseases, such as systemic lupus erythematosus, myositis, and mixed connective tissue disease. A group of individuals without known rheumatic diseases and not receiving immunosuppressive treatment for any other condition served as controls.

### Vaccination

Vaccination against COVID-19 was performed according to the Swedish national vaccination program during 2021–2022. Three different COVID-19 vaccines were used (one adenovirus vector vaccine (ChAdOx1 nCoV-19, AstraZeneca) and two mRNA vaccines (BNT162b2, Pfizer-BioNtech and mRNA-1273, Moderna). The vaccinations were performed using either the same vaccine type or a combination of the two vaccine types according to recommendations from the Swedish authorities (16, 17).

### Data collection

At inclusion, demographic data, diagnosis, time for disease onset, previous and current anti-rheumatic treatments, co-morbidities, physician’s assessment of disease activity, disease activity scores using 28-tender and swollen joint count and erythrocyte sedimentation rate (DAS28ESR) (in patients with arthritis) were recorded. Patients were instructed to take notice of possible side-effects or other unexpected reactions after each vaccine dose as well as the possible effects of vaccination on their rheumatic disease. Patients also reported having a COVID-19 infection and their medical records were searched for this diagnosis. Patients also stated if they had another disease or condition apart from their rheumatic diagnosis (appointed “any comorbidity” in Table 1). Patient-reported outcome measures such as assessment of pain and disease activity were assessed with visual analog scales and physical function measured by health assessment questionnaire was also collected in the Swedish Rheumatology Registry (25). At the same time point, routine blood samples [blood cell count, erythrocyte sedimentation rate (ESR), C-reactive protein, and creatinine] were analyzed. Additionally, the total IgG levels before the last treatment course before the third vaccine dose were measured in rituximab-treated patients (26). Patients were scheduled for a second visit after two vaccinations, as well as after the third and fourth vaccine doses.

### Analysis of antibody response

The levels of IgG antibodies binding to two antigens representing the spike WT (wild-type) protein (spike full-length protein and spike S1 subunit) and one antigen representing the C-terminal fragment of the nucleocapsid protein (used to detect previously SARS-CoV-2 infected individuals) were measured in sera collected pre- and post-vaccination using a multiplex bead-based serology assay (27). Moreover, a set of 12 pre-pandemic samples selected to represent the background distribution were also included in each assay run and used to calculate an antigen and assay-run specific cut-off level for reactivity (27). The cut-off level was calculated as the mean signal intensity of the pre-pandemic samples +6 SD for spike antigens and +12 SD for nucleocapsid antigens (27). A positive antibody response was defined as having antibodies over the cut-off level for both spike antigens (seropositivity) or ≥fourfold increase in post-vaccination antibody levels for both antigens (seroconversion). For omicron subvariants sBA.1 and sBA.2 a positive antibody response was defined as having antibodies over the cut-off level for both antigens.

### Statistical calculations

The geometric mean titer (95% CI) before vaccination, after the third vaccine dose, and the ratio between post-vaccination and pre-vaccination antibody titers were calculated in all groups and compared using the student’s *t*-test. The percentage (%) of responders in each treatment group was compared to the percentage of responders among healthy controls and between responders for spike wild-type antigens compared to responders for omicron subvariants (Chi-squared test). Predictors of antibody response for rituximab-treated patients were determined using logistic regression analysis adjusted for age, gender, diagnosis, IgG before vaccination, rituximab dose before the third vaccine dose, the time between last rituximab dose and the third vaccine dose, prednisolone at the third vaccine dose, and methotrexate at the third vaccine dose. All tests were two-sided and statistical significance was set at 0.05.

Data analyses were performed using SPSS 28 and GraphPad Prism 9.

## RESULTS

In total, 414 patients receiving b/tsDMARDs (283 had arthritis, 75 systemic vasculitis, and 56 other autoimmune diseases) and controls (*n* = 61) entered the study (19). The following treatment groups were included rituximab (*n* = 145), abatacept (*n* = 22), IL6ri (*n* = 79), JAKi (*n* = 58), TNFi (*n* = 68), and IL12/23/17i (*n* = 42) (19). Of these, serum samples were analyzed in 372 patients and 51 controls after two vaccine doses, 370 patients and 52 controls after the third (in detail described in Table 1), and 65 patients and 15 controls after the fourth dose. Four patients switched b/tsDMARD group and four patients terminated their b/tsDMARD treatment during the study period and were not included in the analysis. Methotrexate (MTX) was the most frequent csDMARD, used in 32.7% of the patients. Concomitant prednisolone at dose 3 was used in 137 (37.0%) patients. The median prednisolone dose among the patients on prednisolone was 5 mg (range 1–20 mg). Rituximab was given in combination with at least one csDMARD to 58 patients (66% women; mean disease duration was 18 years and mean age was 67 years). Rituximab with concomitant csDMARD was mostly used in RA/JIA (75.9%) and only in 12.1% of systemic vasculitis patients. Rituximab, abatacept, and IL6r-treated patients were older compared to other treatment groups and controls (Table 1). There were significantly more ever-smokers (*P* = 0.043) and individuals with co-morbidities (*P* < 0.001) among patients compared with the controls (Table 1). To have had a COVID-19 infection as well as having positive nucleocapsid IgG before the second vaccine dose was significantly more common among healthy controls than in patients (both *P* < 0.001). After two or more vaccine doses there was no significant difference in the frequency of COVID-19 infections between patients and controls.

**TABLE 1:** The disease and treatment characteristics at the third vaccine dose

Parameter	Treatment group							
	All patients (*n* = 370)	Rituximab (*n* = 133)	TNF inhibitors (*n* = 61)	IL6 receptor inhibitors (*n* = 71)	Abatacept (*n* = 22)	IL12/23/ IL17 inhibitors (*n* = 27)	JAK- inhibitors (*n* = 56)	Controls (*n* = 52)
Age (median, range years)	60 (22–87)	69 (32–87)	45 (22–63)	66 (32–84)	62 (23–79)	55 (23–75)	53 (24–78)	52 (27–75)
Females (*n*, %)	255 (68.9)	89 (66.9)	43 (70.5)	50 (70.4)	17 (77.3)	12 (44.4)	44 (78.6)	41 (78.8)
Ever smokers (former/current) (*n*, %)	181 (48.9)	75 (56.4)	14 (23.0)	39 (54.9)	13 (59.1)	10 (37.0)	30 (53.6)	12 (23.5) ^*a*^
Disease duration (median, range years)	15.0 (1–61)	13.0 (3–46)	12.5 (1–42)	17.0 (2–61)	22.0 (5–44)	16.5 (4–43)	13.0 (1–52)	
Any comorbidity (*n*, %)	280 (75.7)	126 (94.7)	30 (49.2)	57 (80.3)	16 (72.7)	23 (85.2)	39 (69.6)	16 (31.4) ^*a*^
Diabetes mellitus (*n*, %)	29 (7.8)	19 (14.3)	2 (3.3)	6 (8.5)	1 (4.5)	3 (11.1)	3 (5.4)	1 (2.0)
Hypertension (*n*, %)	85 (23.0)	31 (23.3)	6 (9.8)	21 (29.6)	6 (27.3)	8 (29.6)	13 (23.2)	5 (9.8) ^*a*^
Chronic obstructive lung disease (*n*, %)	8 (2.2)	4 (3.0)	1 (1.6)	4 (5.6)	0	0	0	0
Glucocorticoids (*n*, %)	137 (37.0)	72 (54.1)	6 (10.3)	27 (38.0)	10 (45.5)	1 (3.8)	20 (35.7)	0
Glucocorticoids > 7.5 mg/d (*n*, %)	19 (5.1)	13 (9.8)	0	2 (2.8)	1 (4.5)	0	3 (5.4)	0
csDMARD, any (*n*, %)	170 (45.9)	57 (42.9)	33 (56.9)	28 (39.4)	15 (68.2)	7 (26.9)	18 (32.1)	0
Methotrexate (*n*, %)	121 (32.7)	43 (32.3)	24 (41.4)	22 (31.0)	11 (50.0)	5 (19.2)	16 (28.6)	0
Sulfasalazine, (*n*, %)	15 (4.1)	2 (1.5)	7 (12.1)	4 (5.6)	1 (4.5)	0	1 (1.8)	0
Hydroxychloroquine (*n*, %)	17 (4.6)	11 (8.23	1 (1.7)	3 (4.2)	1 (4.5)	0	1 (1.8)	0
Other csDMARD (*n*, %)	17 (4.6)	10 (7.5)	1 (1.7)	1 (1.4)	2 (9.1)	2 (7.7)	3 (5.4)	0
Type of vaccine								
mRNA vaccine three doses (*n*, %)	309 (83.5)	105 (78.9)	60 (98.4)	49 (69.0)	17 (77.3)	25 (92.6)	51 (91.1)	41 (80.4)
Combination of mRNA and adenovirus vector vaccine (*n*, %)	44 (11.9)	19 (14.3)	0	16 (22.5)	5 (22.7)	1 (3.7)	3 (5.4)	10 (19.6)
COVID-19 infection before vaccination (yes, %)	26 (7.0)	4 (3.0)	2 (3.4)	5 (7.0)	3 (16.7)	7 (29.2)	5 (11.4)	15 (41.7) ^*a*^
Nucleocapsid IgG positive after vaccine dose 2 (*n*, %)	15 (4.1)	4 (3.0)	1 (1.7)	2 (2.8)	1 (5.6)	4 (16.7)	3 (6.8)	10 (27.8) ^*a*^

^*a*^ Significant difference between patients with IRD and immunocompetent controls (Chi2).

Table 1 summarises the demographic, disease and treatment characteristics, smoking status, and comorbidities at the third vaccination dose in the study.

### Antibody response after third and fourth vaccine dose

**Fig 1:**
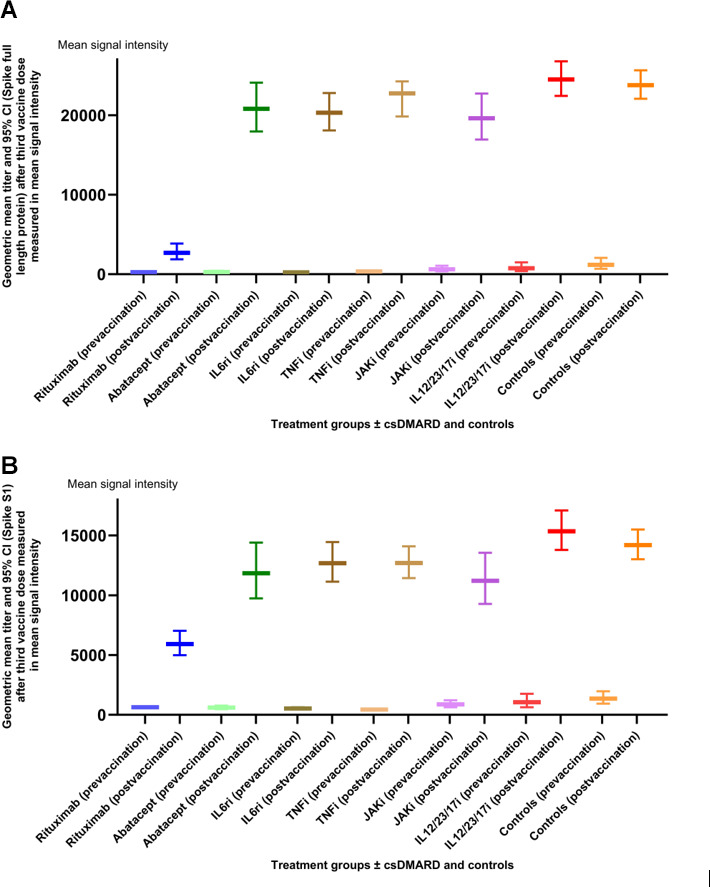
(A and B) Geometric mean titers (95% CI) measured in mean signal intensity before and after the third vaccine dose (4A = spike S1 and 4B = spike full length protein) for each treatment group ±csDMARD and controls. Abbreviation: IL12/23/17i = Interleukin-12/23 and Interleukin-17 inhibitors.

Geometric mean titers (95% CI) before vaccination, after the third vaccine dose, and post-vaccination/pre-vaccination ratio for spike1 and spike full-length protein in different treatment groups and controls are summarised in Table 2. The geometric mean titers (measured in mean signal intensity) before and after the third dose are shown in Fig. 1. Rituximab-treated patients had significantly lower post-vaccination antibody titers as well as post-vaccination/pre-vaccination ratio compared to other groups and controls (*P* < 0.05; student’s *t*-test). Corresponding analysis after the fourth vaccine dose was not performed due to the limited number of samples in some treatment groups.

**TABLE 2:** Pre- and post-vaccination (after the third vaccine dose) geometric mean titer (95% CI) and post-vaccination/pre-vaccination ratio for spike1 and spike full-length protein

Treatment group	Geometric mean titer (95% CI) spike1	Geometric mean titer (95% CI) spike full length protein
All patients		
Pre-vaccination	644 (585–709)	377 (329–432)
Post-vaccination	5,920 (4,985–7,030)	11,120 (9,564–12,929)
Post-vaccination/pre-vaccination ratio	9.2	29.5
Rituximab		
Pre-vaccination	526 (463–598)	251 (215–293)
Post-vaccination	1,104 (743–1,640)	2,692 (1,878–3,860)
Post-vaccination/pre-vaccination ratio	2.1	10.7
TNF inhibitors (TNFi)		
Pre-vaccination	442 (363–539)	356 (276–459)
Post-vaccination	12,699 (11,439–14,098)	24,233 (19,832–22,732)
Post-vaccination/pre-vaccination ratio	28.7	68.1
IL6 receptor inhibitors (IL6ri)		
Pre-vaccination	534 (444–643)	225 (182–279)
Post-vaccination	12,684 (11,130–14,455)	20,304 (18,088–22,792)
Post-vaccination/pre-vaccination ratio	23.8	90.2
Abatacept		
Pre-vaccination	607 (473–779)	274 (172–436)
Post-vaccination	11,846 (9,741–14,406)	20,797 (17,950–24,095)
Post-vaccination/pre-vaccination ratio	19.5	75.9
IL12/23/IL17 inhibitors		
Pre-vaccination	1,064 (644–1,758)	761 (386–1,501)
Post-vaccination	15,349 (13,786–17,088)	24,499 (22,422–26,768)
Post-vaccination/pre-vaccination ratio	14.4	32.2
JAK inhibitors (JAKi)		
Pre-vaccination	884 (637–1,226)	629 (373–1,062)
Post-vaccination	11,211 (9,275–13,552)	19,615 (16,932–22,722)
Post-vaccination/pre-vaccination ratio	12.7	31.2
Controls		
Pre-vaccination	1,357 (937–1,966)	1,177 (675–2,051)
Post-vaccination	14,195 (13,005–15,496)	23,777 (22,052–25,638)
Post-vaccination/pre-vaccination ratio	10.5	20.2

### Proportions of individuals with positive antibody response

**Fig 2:**
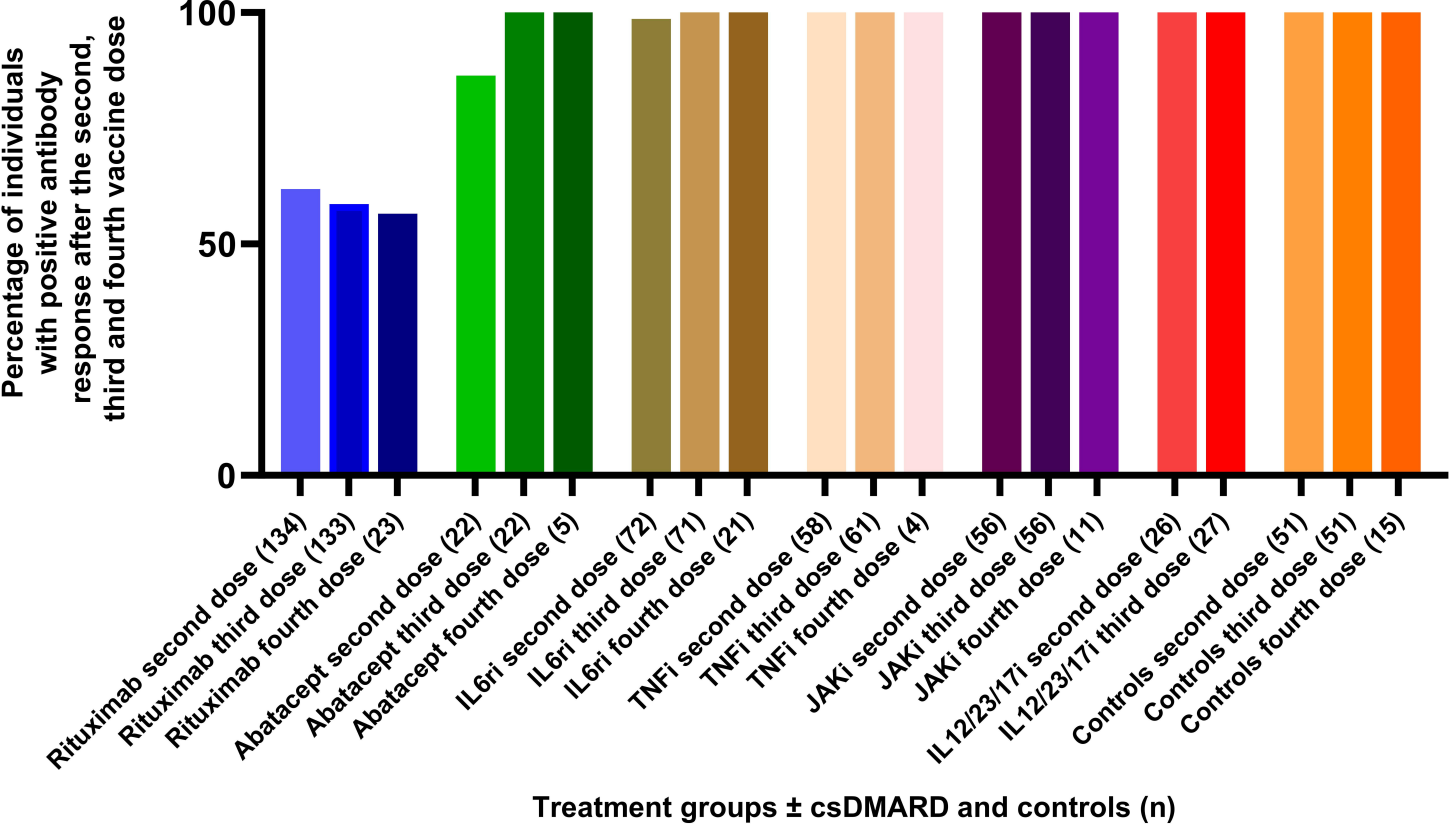
Proportion of participants with positive antibody response [defined as post-vaccination antibody levels over the cut-off (seropositivity) in seronegative patients or ≥fourfold increase in antibody levels] in patients with seropositivity for both spike antigens in each treatment group ±csDMARD and controls after dose two, three, and four.

**Fig 3:**
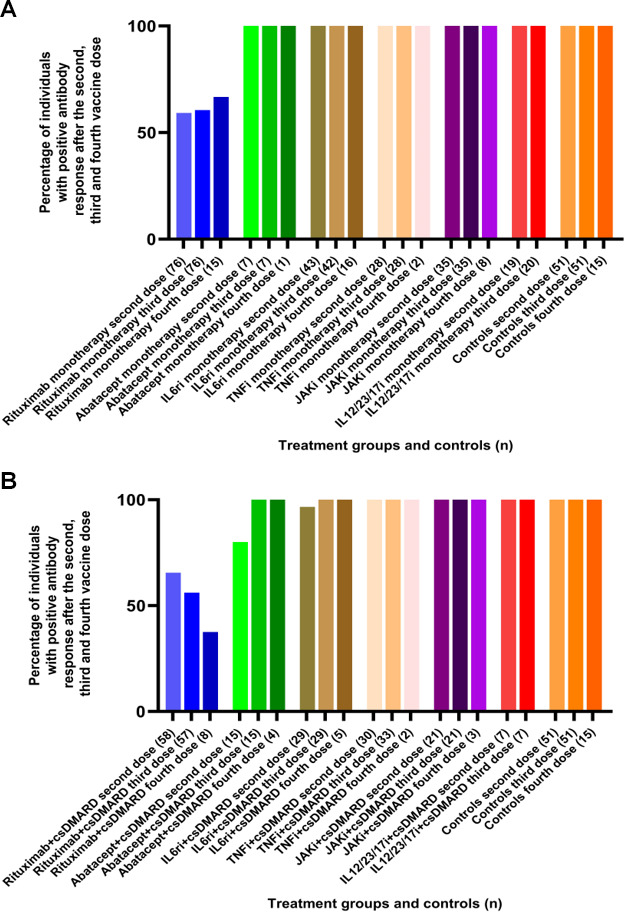
(A and B) Proportion of participants with positive antibody response [defined as post-vaccination antibody levels over the cut-off (seropositivity) in seronegative patients or ≥fourfold increase in antibody levels in patients with seropositivity for both spike antigens] in each treatment group after dose two, three, and four (2A = b/tsDMARDs as monotherapy and 2B = b/tsDMARDs + csDMARD) and controls. (A) The different immunomodulating therapies in monotherapy and controls. (B) The different immunomodulating therapies in combination with csDMARD and controls.

The percentage of responders after two vaccine doses in the abatacept group was significantly lower compared to controls (*P* = 0.025) but increased to 100% after the third vaccine dose. The proportion of patients with positive antibody response after two, three, and four vaccine doses was significantly lower in rituximab patients (62%, 59%, and 57%) compared to controls (100%) (*P* < 0.001). The percentage of responders in all other treatment groups who after two vaccine doses showed a response similar to controls, increased additionally after the third dose and resulted in a positive antibody response in all participants (Fig. 2). In Fig. 3 the treatment is divided into biological therapy as monotherapy (Fig. 3A) and combined with csDMARD (Fig. 3B). We also calculated response rates defined as 10- or 20-fold increase in pre- to post-vaccination antibody titers. As expected, the proportions of individuals with a 10-fold and 20-fold increase in pre-vaccination antibody levels after the third vaccination were lower than those with a fourfold increase in each treatment group but showed the same pattern as a fourfold response, i.e., with rituximab treated patients having the lowest proportion responders.

#### Antibody response after three vaccine doses against omicron subvariants sBA.1 and sBA.2

**Fig 4:**
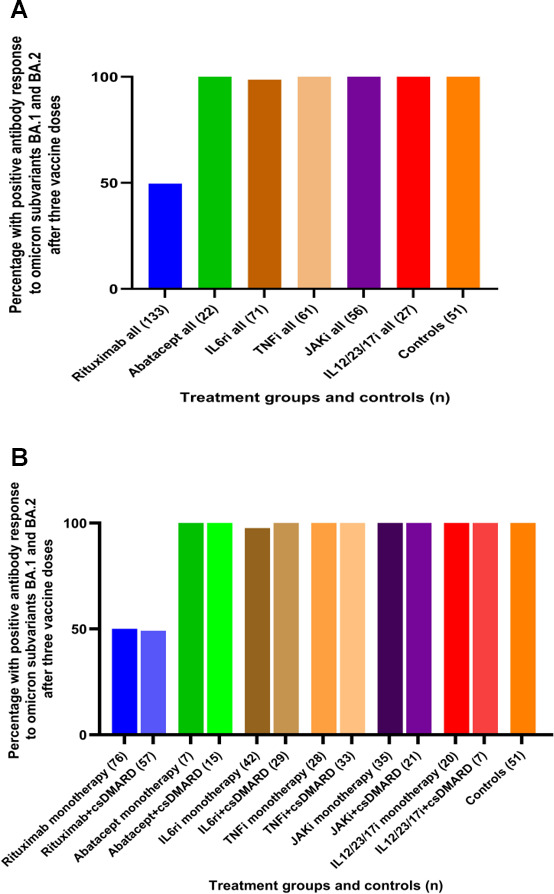
(A and B) Proportion of participants with positive antibody response (defined as having antibodies over the cut-off level for both antigens) after three vaccine doses against omicron subvariants sBA.1 and sBA.2 (3A = b/tsDMARDs ± csDMARD and 3B = b/tsDMARDs as monotherapy and b/tsDMARDs + csDMARD) and controls. (A) The different immunomodulating therapies overall and controls. (B) The different immunomodulating therapies are divided into monotherapy and combination with csDMARD and controls.

Compared to anti-spike wild-type seropositive patients, there were no significant differences in proportions of anti-omicron sBA.1 and sBA.2 seropositive patients in the treatment groups: rituximab (57.6% and 49.3%), IL6ri (both 98.6%), and all other groups (all 100%) (Fig. 4A and B). Compared to controls, sBA.1 and sBA.2 IgG were lower only for the rituximab group (*P* < 0.001) (Fig. 4A and B).

#### Predictors of positive antibody response among rituximab-treated patients

In rituximab patients, the third vaccine dose was administrated after a median of 5 months (range 0–23) following the last rituximab treatment. Table 3 summarizes the results of the analysis of possible predictors of a positive antibody response after the third vaccine dose. Higher total IgG levels before vaccination were associated with better response. Corresponding to data after two vaccine doses, a longer time between the last rituximab course and vaccination was associated with a better response. Concomitant MTX, other DMARDs or concomitant steroids (prednisolone) (yes/no) or daily dose did not have any significant impact on the antibody response.

**TABLE 3:** Predictors of positive antibody response defined as having antibody levels over the cut-off level for both spike antigens or ≥fourfold increase in pre-vaccination antibody levels for both antigens (responders) in patients treated with rituximab^*a*^

Parameter	*P*-value	OR	95% CI
Age (years)	0.942	1.0	0.97–1.03
Gender (female/male)	0.362	0.7	0.3–1.6
Diagnosis (arthritis vs other diagnoses)	0.220	0.5	0.2–1.5
IgG before vaccination	0.032	1.2	1.02–1.4
Rituximab dose before third vaccination (arthritis/vasculitis/inflammatory systemic (1,000 mg vs 500 mg)	0.148	0.5	0.2–1.3
Time between last rituximab dose and third vaccination (months)	0.042	1.4	1.01–1.8
Prednisolone at third vaccination (mg/day)	0.641	1.0	0.9–1.1
Methotrexate at third vaccination (yes/no)	0.672	1.2	0.5–3.2

^*a*^ Adjusted for: age, gender, diagnosis, IgG before vaccination, rituximab dose before the third vaccine dose, time between last rituximab dose and the third vaccine dose, prednisolone at the third vaccine dose, methotrexate at the third vaccine dose.

### Tolerability of the vaccines

No major side effects were seen for any of the vaccines or the different combinations. The most prevalent side effects reported were tiredness, tenderness at the injection site, muscle pain, and headache. Only 17 (5.3%) patients reported a flare in their disease after the third vaccination.

## DISCUSSION

In this multicentre, Swedish study including IRD patients receiving different b/tDMARDs, sufficient immunogenicity after the third and fourth doses of the COVID-19 vaccine was observed in all b/tsDMARDs treatments with or without concomitant DMARD with an exception of rituximab.

In agreement with our previous study, a Norwegian study reported an attenuated response to the standard vaccination regimen in IRD patients compared to healthy controls (19, 22). Furthermore, Benucci et al. demonstrated an increase in antibody production after the third vaccine dose in patients treated with TNFi, JAKi, and IL6ri but not with rituximab which is in agreement with our findings (24).

Jyssum et al. could further show that even if a third vaccine dose did not induce a serological response in rituximab-treated patients with RA, it could boost the cellular immune response (23).

When looking at the non-responders after two vaccines Bitoun et al. could show only a few individuals responding after the third dose independent of time from the last rituximab given (28). In our cohort eight of the 51 rituximab patients without seroconversion after the first two doses were positive responders after three doses and an additional two patients after the fourth vaccine dose. As we reported previously longer time between the last rituximab treatment and vaccination predicted better immunogenicity which was confirmed even for response to the third vaccine dose. This is in line with findings from others (29). Furthermore, higher total IgG before the last rituximab course predicted a better response, probably reflecting the impact of rituximab treatment on the total B cell pool. A Danish study measured B lymphocytes before the third dose and could demonstrate B cells higher than 10/µL to be associated with seroconversion and a conversion rate observed in approximately one-third of the patients, a lower rate compared to our study (30). The number and function of remaining B cells in circulation possibly play a role in antibody response to vaccination, but that analysis was not included in the aims of the present study. In the prediction analysis, concomitant MTX or concomitant corticosteroids did not have any significant impact on the antibody response.

Studies of four or further vaccination doses in patients with IRD and different immunomodulating therapies are sparse and need further investigation (29, 31).

IgG antibody levels to omicron spike subvariants sBA.1 and sBA.2 were similar to the levels of IgG anti-spike wild-type (full-length spike and S1). All treatment groups with the exception of rituximab developed satisfactory responses to both spike subvariants. The absence of difference in the IgG response rate to spike wild-type and subvariants could be partly due to the fact that our serology assay is based on full-length proteins while the site of variation involves a smaller portion of the protein sequence; therefore, the IgG polyclonal response toward spike full-length protein could mask possible differences related to antibody binding to specific sites (32).

Antibody levels have been shown to be correlated to the degree of clinical protection against COVID-19 infection (33). Our results indicate that three vaccine doses probably provide protection against omicron virus subvariants for a majority of patients, with the exception of patients treated with rituximab.

In accordance with other studies, we found the third vaccine dose to be safe, with no major side effects (23, 24, 34). Contrary to Syversen et al. who reported a relatively high percentage (16%) of the patients getting a flare after dosage three (whereas only 6% after doses 1 and 2, respectively) only 5.3% of the patients in our cohort reported a worsening in their rheumatic disease after the third vaccination, in comparison with 3.4% after two doses (22).

The strengths of this study are the well-characterized patient population with a wide range of autoimmune rheumatic diseases and immunosuppressive treatments as well as the prospective design. The longitudinal follow-up in a real-life setting gives a generalizability for the results. Furthermore, we provide the data on the serological immunity against the omicron virus subvariants.

A limitation of this study is that cellular immune response has not been measured, although such analyses are currently ongoing. Further studies are needed to establish if the serologic responses are predictive for severe COVID-19 infection and at what level “sufficient” protection is settled. Another limitation is the low number of patients in some of the treatment groups.

### Conclusion

In this Swedish, multicentre study including IRD patients receiving different b/tsDMARDs, a sufficient immunogenicity of the third and fourth doses of the COVID-19 vaccine was observed in all treatment groups with an exception of patients treated with rituximab. The increased proportion of positive antibody response among some rituximab patients not responding to two vaccine doses along with sufficient serological response in other immunosuppressed patients, including response to the omicron subvariants, supports the current recommendations on additional vaccine doses.

## Data Availability

Raw data are available in the supplemental material.

## SUPPLEMENTAL MATERIAL

Supplemental material spectrum.02981-23-s0001.xlsx Raw data.

ASM does not own the copyrights to Supplemental Material that may be linked to, or accessed through, an article. The authors have granted ASM a non-exclusive, world-wide license to publish the Supplemental Material files. Please contact the corresponding author directly for reuse.
